# Lymph Node Yield and Ratio in Selective and Modified Radical Neck Dissection in Head and Neck Cancer—Impact on Oncological Outcome

**DOI:** 10.3390/cancers13092205

**Published:** 2021-05-04

**Authors:** Sean C. Sheppard, Lukas Frech, Roland Giger, Lluís Nisa

**Affiliations:** Department of Otorhinolaryngology-Head and Neck Surgery, Inselspital, Bern University Hospital, University of Bern, 3010 Bern, Switzerland; lukas.frech@students.unibe.ch (L.F.); lluis.nisa@dbmr.unibe.ch (L.N.)

**Keywords:** neck dissection, selective neck dissection, radical modified neck dissection, head and neck cancer, squamous cell carcinoma, chemoradiation, lymph node yield, lymph node ratio, lymph node density, oncological outcome

## Abstract

**Simple Summary:**

Head and neck cancers metastasize into the neck lymph nodes. The surgical removal of neck lymph nodes (i.e., neck dissection) is commonly performed before or after radiation therapy. The most common types of neck dissection include selective (with removal of nodes at risk) and radical modified (with removal of all neck lymph nodes). Given the variability in surgical techniques, quality and prognostic markers for neck dissection are missing. Lymph node ratio (LNR: number of metastatic nodes/total number of nodes harvested) has been previously suggested as a quality and prognostic marker. Here, we assess the impact of prior irradiation on the lymph node yield (LNY) as well as the role of LNR in both selective and modified radical neck dissection. We found that previous irradiation to the neck leads to a reduced LNY; there is no change in LNR. LNR has a prognostic impact in modified radical neck dissection but not in selective neck dissection.

**Abstract:**

*Background*: Lymph node metastases are associated with poor prognosis in head and neck squamous cell carcinoma (HNSCC). Neck dissection (ND) is often performed prior to or after (chemo)radiation (CRT) and is an integral part of HNSCC treatment strategies. The impact of CRT delivered prior to ND on lymph node yield (LNY) and lymph node ratio (LNR) has not been comprehensively investigated. *Material and methods:* A retrospective cohort study was conducted from January 2014 to 30 June 2019 at the University Hospital of Bern, Switzerland. We included 252 patients with primary HNSCC who underwent NDs either before or after CRT. LNY and LNR were compared in patients undergoing ND prior to or after CRT. A total of 137 and 115 patients underwent modified radical ND (levels I to V) and selective ND, respectively. The impact of several features on survival and disease control was assessed. *Results:* Of the included patients, 170 were male and 82 were females. There were 141 primaries from the oral cavity, 55 from the oropharynx, and 28 from the larynx. ND specimens showed a pN0 stage in 105 patients and pN+ in 147. LNY, but LNR was not significantly higher in patients undergoing upfront ND than in those after CRT (median: 38 vs. 22, *p* < 0.0001). Cox hazard ratio regression showed that an LNR ≥ 6.5% correlated with poor overall (HR 2.42, CI 1.12-4.89, *p* = 0.014) and disease-free survival (HR 3.416, CI 1.54-754, *p* = 0.003) in MRND. *Conclusion:* ND after CRT leads to significantly reduced LNY. An LNR ≥6.5% is an independent risk factor for decreased overall, disease-free, and distant metastasis-free survival for MRND.

## 1. Introduction

Lymph node metastases (LNMs) are common at the time of diagnosis in head and neck squamous cell carcinomas (HNSCCs), while the rate of occult LNMs is up to 20% in some subtypes of HNSCC [1,2,3,4,5]. The presence of LNMs bears a major prognostic influence, with an estimated 50% reduction in overall survival in nodal negative vs. nodal positive HPV-negative HNSCC [6,7,8]. Features such as number, size, location in the neck, and the presence of extracapsular extension (ECE) among others equally have a negative impact on survival and oncological outcome [9,10,11,12].

Management strategies for the neck encompass primary radiotherapy/chemoradiation (CRT), neck dissection (ND), or combinations of these treatment modalities. Neck dissection can be performed upfront prior to CRT of the primary tumor +/− the neck or following failed primary CRT as surgical salvage treatment. The extent of ND is variable and depends on factors such as clinical involvement of neck LNs, site of the primary tumor, and prior therapy, among others [13]. From a surgical perspective, ND is basically classified in selective ND (SND) when only some nodal groups are removed, and radical or modified radical ND (MRND) when levels I to V, with or without other structures such as the sternocleidomastoid muscle, the accessory nerve, or the internal jugular vein are removed [14].

Given that surgical technique can vary from center to center, standard quality markers for ND that correlate with outcome have not been comprehensively explored in the setting of upfront and salvage ND. A number of recent studies analyzed the relevance of parameters such as total number of LNs removed or lymph node yield (LNY), and as well as lymph node ratio (LNR), which is defined as the ratio between metastatic and total LNs in a ND specimen. Moreover, the effect of CRT on LNY has not been assessed in large cohorts of patients with HNSCC undergoing ND. While the importance of LNY and LNR is established in human malignancies such as colorectal and breast cancer, no general consensus on a specific yield or ratio has been definitively established in head and neck squamous cell carcinoma (HNSCC) [15,16,17,18,19,20,21,22].

The primary purpose of this study is to assess the influence of CRT on LNY/LNR as well as the impact of LNR in terms of survival and disease control in patients undergoing SND and MRND.

## 2. Materials and Methods

### 2.1. Ethical Issues

Approval was obtained from our ethics committee (Cantonal Ethics Committee of Bern—reference number: KEK 2018-01735). All patients included in this study were treated at Bern University Hospital (Inselspital), Bern, Switzerland. Patients’ data were stored anonymously in a database only accessible to the principal investigators of this study.

### 2.2. Study Design and Patient Eligibility

The study cohort consisted of patients diagnosed with HNSCC who underwent ND with curative intent between 1 January 2014 and 30 June 2019 at the University Hospital and Cancer Institute of Bern, Switzerland. Patients’ details have been standardized in a specific database since 1 January 2014. All management approaches were discussed at a multidisciplinary tumor board. Inclusion criteria were (1) histologically-proven SCC of the oral cavity, oropharynx, larynx, or hypopharynx, as well as LNMs of a SCC of unknown primary (CUP); (2) management of neck with ND (either alone, prior or following CRT, regardless of primary tumor treatment). Patients were excluded in the following cases: (1) histopathology other than SCC; (2) head and neck cancer affecting other sites than mentioned above; (3) ND and histopathological evaluation performed at external institutions; (4) previous ipsilateral ND; and (5) insufficient data.

### 2.3. Clinical Data

Patient demographics (age, gender) and clinical data (site of primary, TNM stage, global UICC 8th classification, type of CRT, surgical and histopathological reports) were collected in a study-specific database. The process of data extraction was double-checked by two of the authors (S.C.S and L.N).

### 2.4. Histopathological Review

Neck dissection specimens were either sent en bloc or divided into nodal levels by the surgeon. In case of en bloc resections, non-specified levels are classified as “non-defined”. The standard histopathological diagnosis at our institution has been previously reported [9].

### 2.5. Data Analysis

Statistical analysis was performed using the SPSS statistics software Version 25 (IBM, Armonk, NY, USA) and with GraphPad Prism 9 (GraphPad Software, Inc., San Diego, CA, USA). Lymph node yield refers to the actual number of LNs removed. Lymph node ratio was defined as the number of LNMs divided by the total of LNs excised, regardless of the extent of the ND. The value was reported as a percentage. First, we considered all included NDs, and analyzed LNY and LNR per type of ND, per side, and per level. Modified radical neck dissections was defined as NDs of levels I to V with or without removal of other muscular, nervous, or vascular structures (both radical and modified radical NDs are included in the abbreviation MRND). Then, subgroup comparisons were performed between SND I and III (the most commonly performed) and MRND. For bilateral ND consisting of an SND on one side and an MRND on the other side, only the MRND side was evaluated. In cases of bilateral NDs with metastases in only one side of the neck, only the pathologically involved side was used for the analysis. In case of bilateral involvement or bilateral absence of metastases, the median value for both sides was used. The cutoff value of LNR was based on the minimum *p*-value approach. For all survival analyses, the date of ND was considered the beginning point. We used the unpaired *t*-test to compare clinical features, LNY and LNR prior to and after CRT. Survival curves were plotted using the Kaplan–Meier method and differences in survival were plotted using the log-rank test and Cox proportional hazard to obtain univariate and multivariate hazard ratios (HRs), respectively. For multivariate analyses and to avoid missing potentially relevant variables, we considered variables present in at least 10% of the patients and that had a *p*-value < 0.1 in the log-rank test analysis. Variables fulfilling these criteria were entered in the Cox’s proportional-hazards model. LNRs was included regardless of significance in both the SND and the MRND groups. Only variables with a *p*-value < 0.05 in the final model were considered to be significant independent risk factors. All tests were two-sided, and a *p*-value < 0.05 was deemed to denote significance.

## 3. Results

### 3.1. Patients’ Demographics and Treatment Features

The study cohort consisted of 252 patients fulfilling the inclusion criteria, who underwent 169 unilateral ND and 83 bilateral ND for a total of 335 sides operated (Table 1). Most patients were male (170, 67.46%) and median age at diagnosis was 62.85 years (range 24.9–95.1 years). Oral cavity and oropharynx were the primary sites in 78.17%. Seventeen patients (6.75%) had a CUP. Dichotomized nodal stage in N0 and N+ was 110 cN0/142 cN+ and 105 pN0/147 pN+. Stage III–IV disease was diagnosed in 57.94% of the patients. Treatment modality of the primary tumor was surgical for 189 patients and non-surgical for 28 patients. Ninety-nine (39.29%) patients underwent adjuvant CRT of the neck. No statistically significant difference between demographics and clinical features in patients undergoing upfront vs. salvage ND was found (Appendix A, Table A1).

**Figure 1:**
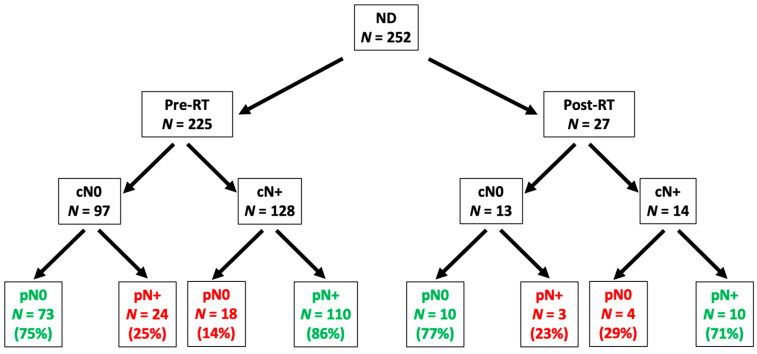
Workflow showing clinical and pathological status in 252 patients undergoing neck dissection.

One hundred and eighty-one were SNDs of different types and 154 were MRND. The vast majority of NDs took place prior to CRT (89.29% upfront vs. 10.71%). In the upfront neck dissection group, 56.89% were patients were cN+ vs. 51.85% in the post-irradiation group. The rate of occult metastases (cN0/pN+) was 25% in the pre-irradiation group and 23% in the post-irradiation group, while the concordance between cN+ and pN+ cases was higher in the pre-irradiation than in the post-irradiation group (86% vs. 71%, respectively) (Figure 1).

### 3.2. Radiation Induces Reduction of Lymph Node Yield but Not Lymph Node Ratio

We determined LNY and LNR in patients undergoing ND before and after CRT (Table 2). LNY and LNR were compared using all sides, then comparing SND vs. MRND, and finally per nodal level. LNY was significantly higher patients undergoing upfront ND than in those undergoing salvage ND (median: 38 vs. 22, *p* < 0.0001), with a higher level of significance in MRND. The differences between upfront and salvage ND were significant for all nodal levels, except for level V, which is the level with the lowest LNY.

In contrast to the LNY, we could not find a statistically significant difference in terms of LNR in upfront vs. salvage ND (mean: 3.72% vs. 2.85%, *p* = 0.5788), but comparison of LNR in SND vs. MRND yielded a highly significant difference (1.40 ± 0.42 vs. 6.16 ± 1.0, *p* < 0.0001).

### 3.3. Survival and Disease Control

We assessed the survival and disease control of the 252 patients included in the cohort. Median follow-up from the time of treatment to the last oncological visit or event was 37 months (range 0–12.42 years) and of 43 months (range 2–149 months) for surviving patients. Overall survival was 81.02% (95% CI, 75.48–85.43%) at 2 years and 61.89% (95% CI, 53.23–69.42%) at 5 years. Disease-free survival (DFS) was 78.82% (95% CI, 72.96–83.56%) at 2 years and 72.60% (95% CI, 65.99–71.14%) at 5 years. Regional recurrence free-survival at 2 years in 89.03% (95% CI, 84.19–92.46%) and at 5 years in 84.94% (95% CI, 78.99–89.32%). Distant-metastasis free-survival was obtained for an estimated 88.46% (95% CI, 83.49–92.00%) at 2 years and for 87.26% (95% CI, 82.02–91.05%) at 5 years.

### 3.4. Survival Outcome in SND and MRND

For the analysis of comparative survival outcomes, we separately evaluated SND (*n* = 73) and MRND (*n* = 137) because of their different indications. To ensure homogeneity in the SND group, we included only SND of level I to III. Their clinical and pathological features are shown in Table 1. We selected a cutoff LNR of 6.5% based on the minimum *p*-value.

The SND group consisted exclusively of cN0 oral and oropharynx SCC (Table 1). In the univariate analysis, after dichotomizing clinical and pathological features, age over 65 years and advanced T-stage (T3–4) were associated with a worse OS with statistical significance. For DFS, advanced T-stage (T3–4), pN+, and presence of extracapsular extension had worse disease control. Regional control and distant metastasis-free survival (DMFS) showed no statistically significant association with clinical features. LNR did not show a correlation with survival outcome. In the multivariate analysis (Appendix B, Table A2), Cox hazard ratio showed advanced age as well as advanced T-stage (T3–4) as independent predictors of poorer OS.

For MRND, univariate analysis was reported in Appendix C, Table A4. OS was adversely affected in the older age group, advanced T-stage, HPV negative tumors, and LNR of ≥6.5% with a statistically significant difference. Patients who were longer free of recurrence had lower T-stage, pN0, HPV positive, LNR < 6.5% and had a surgical treatment of the primary. For regional control, male, pN0, and surgical treatment of the primary showed a better control. DMFS was worse for advanced T-stage, pN+, and LNR ≥ 6.5%.

Upon multivariate analysis (Table 3), LNR ≥ 6.5% and advanced T-stage was correlated with poor OS, DFS, and DMFS. The presence of LNMs had a worse DFS.

## 4. Discussion

In this study, we evaluated the impact of CRT on LNY and LNR in patients with HNSCC managed by neck dissection, as well as the prognostic impact of LNR in SND and MRND. The main findings are that (1) previous CRT is associated with a significant decrease of LNY in ND specimens and in all nodal levels except level V, (2) previous CRT does not affect LNR, and (3) a lymph node ratio ≥6.5% is an independent predictor of decreased OS, DFS and DMFS in patients undergoing MRND but not in those with SND I-III.

The 300 lymph nodes contained within the fatty and connective tissue of the neck represent the first metastatic target of head and neck malignancies. As such, management of the neck is an integral part of HNSCC management [14,23]. In its original description, ND encompassed the removal of all nodal levels of the neck along with the sternocleidomastoid muscle, the internal jugular vein, and the accessory nerve. Given the morbidity of such an approach and the fact that HNSCC metastasizes to the neck in predictable patterns, several modifications of ND have been introduced over the last 30 years. Therefore, the extent of neck surgery is dependent on the clinically and pathologically assessed nodal status and the site of the primary tumor [24]. MRND and SND currently represent the standard approaches to the clinically positive and negative neck, respectively [25].

CRT is both an alternative approach to ND and a complementary strategy. CRT leads to tissular changes including fibrosis and atrophy of soft tissues, including the lymph nodes. RT has been shown to destroy the LN parenchyma, changing its size and number [26]. LNY has been extensively researched in other malignancies such as colon and rectal cancers, where the extent of lymphadenectomy is associated with improved survival outcome [15,17,22,27,28,29].

Our study shows a significant decrease in nodal yield in ND after CRT, from a median nodal yield of 38 to 22 LNs, except in level V. In line with this, several studies have demonstrated a reduction in global LNY in patients with oral, oropharyngeal, nasopharyngeal, laryngeal, hypopharyngeal, and upper esophageal malignancies undergoing preoperative CRT [30,31,32,33]. Yu et al. [34] recently published a large study cohort of 1024 ND showing an average decrease of 7.5 nodal yield in patients undergoing MRND after CRT. Additionally, older age was correlated with a lower LNY. Lippert et al. [35] reported a lower LNY equally in clinically N+ patients undergoing salvage ND, along with a lower prevalence of ECE. An analysis of a Surveillance, Epidemiology, and End Results (SEER) database by Flowstone et al. [33] found a mean decrease of 7.8 in LNY (from 29.7 to 21.9). It is noteworthy that a number of studies were not able to show a significant decrease of LNY post-CRT [30,36].

In their landmark study, Ebrahimi et al. [12] suggested a minimal LN harvest of 18 for patients undergoing SND in oral SCC as a quality marker, as this LNY threshold was shown to be an independent predictor of OS, DSS, and DFS. This data were prospectively validated by two NRG oncology trials reporting an improved survival and decreased loco-regional failure taking 18 as a threshold for LNY [37]. None of these studies included patients undergoing ND after CRT. In our study, we found a median LNY of 38 in upfront ND and 22 in salvage ND and no prognostic impact using 18 as a threshold. This divergence can be easily explained by the fact that there is significant inter-institution and inter-individual variability in terms of surgical technique, as well as significant variability related to histopathological evaluation. All these factors certainly contribute to hinder the standardization of LNY as a prognostic marker [38,39,40,41].

Therefore, we suggest that LNR is a more accurate surrogate for disease extent and may bear a better correlation with surgical outcomes, along with the standard TNM staging. LNRs varying between 1% and 20% have been reported to have a prognostic impact in HNSCC with a general consensus that higher LNRs are associated with a poorer outcome [11,38,42]. In this study, we compared the impact of LNR as a prognostic factor in SND and MRND. We found an LNR of 6.5% or superior to be an independent prognostic factor for decreased OS, DFS, and DFMS in patients managed with MRND but not in those undergoing SND. Given that SND is indicated in patients with clinically negative (cN0) necks, it is not surprising that this threshold may be too high for SND. The incidence of occult neck metastases was 23% in patients with SND, and consequently, almost 80% of patients in this group had an LNR of 0. We found that the threshold for LNR needed to be reduced to under 3.5% to bear prognostic significance in patients with SND (data not shown). LNR is obviously a less meaningful marker in patients with clinically node negative necks, in which markers such as LNY provide a qualitative measure that is probably more relevant in diagnostic terms [12].

Despite being a pertinent prognostic marker, LNR has also its limitations. Having LNY as the denominator, a higher yield will cause a decrease of LNR. For example, Marres et al. [43] showed a decrease of LNR through the increase of LNY with the addition of pathology technicians involved in the work-up of ND specimens. In their study, level V showed the least LNs before changing the histopathological technique of diagnosis, and we discuss that the level V nodes are more difficult to identify. Our study showed a lower nodal yield in the level V with no significant change before or after CRT. With different lymph drainage, the level V has a less important role in HNSCC and is reported to have lower metastasis rates [44]. Given that SND has successively gained more importance with improved imaging-based staging, some authors have suggested adapting LNR cutoffs to the type of ND or levels removed [11]. Liao et al. [45] reported an LNR of 16% for level I–III ND and 4.8% for MRND in pN+ OSCC. Concordant to these results, our findings suggest that different LNR correlated to the extent of ND are suitable for the evaluation of oncological outcome.

In terms of prognosis, an SEER database analysis by Roberts et al. [46], higher LNRs, and pN stage demonstrated poorer OS. However, their multivariate analysis revealed only pN stage to be an independent variable. In contrast, a multi-institutional study of 11 cancer centers including 4254 patients by Patel et al. [20] found an LNR of 7, similar to our threshold of 6.5%, to be a superior prognosticator than N-staging for OSCC. We hypothesize in line with previous studies that LNR is an easy-to-implement feature in future TNM staging systems. Features such as ECE were added in the last UICC/AJCC edition. ECE is a more variable and difficult to assess histological feature than LNR, which is therefore possibly more consistent and possibly a superior prognosticator than ECE [9,47]. An important question to address within the frame of prospective multicentric trials would be to assess whether an LNR > 6.5% warrants postoperative chemoradiation, independent of ECE status.

We need to acknowledge a certain number of limitations in this study, primarily its retrospective design and the relatively small size of the subgroup of patients undergoing ND after CRT. Moreover, as discussed above, our study suffers the usual variability related to surgical technique and level of pathologist training. The fact that our study takes place in a single University Cancer Institute with standardized procedures reduces this variability. Given the limitations in using LNY mentioned, larger, multicentric, and prospective studies would be desirable.

## 5. Conclusions

The major finding is that an LNR of 6.5% or superior is an independent predictor of survival for OS, DFS, and distant metastasis control for MRND but not for SND. In addition, chemoradiation decreases the nodal yield in all levels, except level V. Furthermore, LNR would be an easy feature to integrate in the UICC/AJCC cancer staging, helping in the prognostic stratification of patients.

## Figures and Tables

**Table 1:** Clinical and pathological features. *n* = 252.

Features	Variables	SND I–III * (*n* = 73)	MRND (*n* = 137)	Total (*n* = 252)
Gender	Male	42	91	170
	Female	31	46	82
Age (Years)	≥65	35	53	108
	<65	38	84	144
Primary Site	Oral	71	61	141
	Oropharynx	2	42	56
	Hypopharynx	-	9	10
	Larynx	-	10	28
	CUP	-	15	17
T stage ^1^	T0	-	30	35
	T1–2	62	67	151
	T3–4	11	40	66
cN stage	cN0	73	5	110
	cN+	-	132	142
pN stage	pN0	56	23	105
	pN+	17	114	147
ECE	Present	2	83	93
	Absent	71	54	159
HPV	Positive	2	22	28
	Negative	71	98	203
	Unknown	-	17	21
Overall 8th UICC Stage (pathological)	I–II	53	35	106
	III–IV	20	102	146
Treatment of primary	Surgery only	61	26	111
	Surgery + CRT	12	56	78
	CRT only	-	25	28
	None	-	30	35
Treatment of the Neck (ND)	Surgery only	69	53	153
	Surgery + CRT	4	84	99
Previous CRT	Upfront ND	70	123	225
	Salvage ND after CRT	3	14	27

^1^ T stage: pathological stage (pT) was used when available. T0 = 35 cases of neck metastases of a carcinoma of unknown primary (cT0) or exclusively regional recurrences (rcT0). Abbreviations: CUP—carcinoma of unknown primary, ECE—extracapsular extension, HPV—human papillomavirus, CRT—(chemo)radiation, ND—neck dissection, MRND—radical or modified radical neck dissections, SND—selective neck dissection, w/o—without. * Note that only SND level I–III were included.

**Table 2:** Lymph node yield and lymph node ratio prior and after CRT. *n* = 335 specimens.

Neck Dissection Features	Total	Upfront ND	Salvage ND after CRT	*p* Value
Lymph node yield median (IQR, min, max)				
Overall, *n* = 335	37 (20, 6, 97)	38 (19, 6, 97)	22 (12, 6, 48)	<0.0001
MRND, *n* = 154	45 (17, 6, 97)	46 (15, 6, 97)	21 (12, 16, 48)	<0.0001
SND level I-III, *n* = 119	31 (15, 9, 72)	31 (16, 9, 72)	15 (14, 10, 38)	0.0405
SND level II-IV, *n* = 38	28.5 (17, 6, 69)	36 (22, 13, 69)	24.5 (8, 6, 33)	0.0039
By Levels:				
Level I, *n* = 253	5 (4, 1, 14)	5 (4, 1, 14)	3 (2.5, 1, 9)	0.0305
Level II, *n* = 303	14 (11, 1, 46)	15 (12, 1, 46)	6 (6.5, 1, 23)	<0.0001
Level III, *n* = 292	9 (7, 1, 30)	9 (6, 1, 30)	5 (3.5, 1, 19)	0.0001
Level IV, *n* = 161	8 (8, 1, 29)	9 (7, 1, 29)	6 (4.5, 1, 15)	0.0145
Level V, *n* = 133	6 (6, 1, 38)	6 (6, 1, 38)	5.5 (5, 1, 15)	0.2493
No defined Level, *n* = 122	7.5 (15, 1, 86)	9 (19, 1, 86)	4 (5, 1, 13)	0.0302
Lymph node ratio in % mean (IQR, min, max)				
Overall, *n* = 335	3.62 (4.08, 0, 83.33)	3.72 (4.96, 0, 83.3)	2.85 (5.26, 0, 20)	0.5788
MRND, *n* = 154	6.16 (5.03, 0, 83.33)	6.40 (4.76, 0, 83.33)	3.89 (6.25, 0, 10)	0.4269
SND level I–III, *n* = 119	1.40 (0, 0, 28.57)	1.27 (0, 0, 28.57)	5 (10, 0, 20)	0.0618
SND level II–IV, *n* = 38	0.47 (0, 0, 8)	0.635 (0, 0, 8)	0 (0,0)	0.2785
By Levels:				
Level I, *n* = 253	3.21 (0, 0, 85.71)	2.88 (0, 0, 85.71)	7.08 (0, 0, 66.67)	0.0759
Level II, *n* = 303	4.53 (5, 0, 100)	4.52 (5.26, 0, 100)	4.64 (0, 0, 50)	0.9551
Level III, *n* = 292	2.89 (0, 0, 75)	2.95 (0, 0, 75)	2.30 (0, 0, 66.66)	0.7428
Level IV, *n* = 161	2.05 (0, 0, 100)	2.28 (0, 0, 100)	0.69 (0, 0, 16.66)	0.5428
Level V, *n* = 133	0.98 (0, 0, 66.66)	1.10 (0, 0, 66.66)	0 (0,0,0)	0.5806
No defined Level, *n* = 122	12.06 (8.33, 0, 100)	12.11 (9.10, 0, 100)	11.74 (8.33, 0, 100)	0.9593

Abbreviations: IQR—interquartile range, CRT—(chemo)radiation, ND—neck dissection, MRND –modified radical neck dissections, SND—selective neck dissection, w/o—without.

**Table 3:** Multivariate analysis of modified radical neck dissection factors associated with survival. *n* = 137.

Features	Variables	OS		DFS		Regional Control		DMFS	
		HR (95%CI)	*p*-Value	HR (95%CI)	*p*-Value	HR (95% CI)	*p*-Value	HR (95% CI)	*p*-Value
Gender	Male vs. Female						n.s.		
Age (years)			n.s.						
T stage ^1^	T1–2 vs. T3–4	3.488 (1.723–7.06)	0.001	3.45 (1.568–7.591)	0.002			6.109 (2.069–18.035)	0.001
pN stage	pN0 vs. pN+			8.434 (1.051–67.71)	0.045		n.s.		n.s.
ECE	ECE+ vs. ECE-				n.s.				
HPV ^2^	HPV+ vs. HPV-		n.s.		n.s.				n.s.
Therapy of the Primary ^2^	Surgery vs. CRT				n.s.		n.s.		n.s.
LNR	≥6.5% vs. <6.5%	2.418 (1.196–4.885)	0.014	3.407 (1.539–7.544)	0.003		n.s.	3.085 (1.152–8.266)	0.025

^1^ Patients with T0 were excluded from the analysis. ^2^ Patients with unknown HPV status were excluded from the analysis. Abbreviations: OS—overall survival, DFS—disease-free survival, DMFS—distant metastasis-free survival, ECE—extracapsular extension, HPV—human papillomavirus, CRT—(chemo)radiation, LNR—lymph node ratio, HR—hazard ratio, CI—confidence interval, n.s.—non significant.

## Data Availability

The data presented in this study are available on request from the corresponding author. Institutional permission will need to be sought to share data with external parties.

## Appendix A

**Table A1:** Patients clinical and pathological features prior and after CRT. N = 252.

Features		Total	Upfront ND (*n* = 225)	Salvage ND after CRT (*n* = 27)	*p* Value
Gender					0.9262
	Male	170	152	18	
	Female	82	73	9	
Age (years)					0.3195
	≥65	108	94	14	
	<65	144	131	13	
T stage ^1^					0.401
	T0	35	23	12	
	T1–2	151	143	8	
	T3–4	66	59	7	
cN Stage					0.6197
	cN0	110	97	13	
	cN+	142	128	14	
pN stage					0.2577
	pN0	105	91	14	
	pN+	147	134	13	
ECE	Present	93	83	10	0.9880
	Absent	159	142	17	
HPV ^2^	Positive	28	26	2	0.5501
	Negative	203	181	22	
	Unknown	21	18	3	
Overall 8th UICC-Stage (pathological)					0.8834
	I-II	106	95	11	
	III-IV	146	130	16	
Treatment of primary ^3^					0.1235
	Surgery only	111	98	13	
	Surgery + CRT	78	76	2	
	CRT only	28	28	0	
	None	35	23	12	
Treatment of neck (ND)					<0.0001
	Surgery only	153	126	27	
	Surgery + CRT	99	99	0	

^1^ Patients with T0 were excluded from this analysis. ^2^ Patients with unknown HPV status were excluded from this analysis. ^3^ Surgical vs. non-surgical therapy of the primary was analyzed; cases of carcinoma of unknown primary (cT0) or isolated regional recurrences (rcT0) were excluded from this analysis. Abbreviations: ND—neck dissection, ECE—extracapsular extension, HPV—human papillomavirus, CRT—(chemo)radiation, w/o—without.

## Appendix B

**Table A2:** Univariate analysis of selective I–III neck dissection * factors associated with survival. *N* = 73.

Features	Variables	OS	DFS	Regional Control	DMFS
Gender	male vs. female	0.243	0.924	0.176	0.369
Age (years)	<65 vs. ≥65	0.006	0.546	0.230	0.369
T-Stage ^1^	T1–2 vs. T3–4	0.001	0.046	0.382	0.728
pN-Stage	pN0 vs. pN+	0.139	0.018	0.485	0.617
ECE	ECE+ vs. ECE-	0.734	0.002	0.862	0.901
HPV ^2^	HPV+ vs. HPV-	0.687	0.696	0.805	0.901
Previous CRT	Upfront ND vs. Salvage ND after CRT	0.559	0.226	0.720	0.826
Therapy of Neck (ND)	surgery vs. surgery + CRT	0.484	0.223	0.720	0.859
LNR	≥6.5% vs. <6.5%	0.533	0.197	0.740	0.826

^1^ Patients with T0 were excluded from this analysis. ^2^ Patients with unknown HPV status were excluded from this analysis. Abbreviations: OS—overall survival, DFS—disease-free survival, DMFS—distant metastasis-free survival, ECE—extracapsular extension, HPV—human papillomavirus, CRT—(chemo)radiation, LNR—lymph node ratio. * Note that only SND levels I–III were included.

**Table A3:** Multivariate analysis of neck dissection factors associated with survival in patients with selective neck dissection level I–III *. *n* = 73.

Features	Variables	OS		DFS	
		HR (95% CI)	*p*-Value	HR (95% CI)	*p*-Value
Age (years)		1.111 (1.045–1.181)	0.001		
T stage ^1^	T1–2 vs. T3–4	3.772 (1.104–12.892)	0.034		n.s.
pN stage	pN0 vs. pN+				n.s.
ECE	ECE+ vs. ECE-				n.s.
LNR	≥6.5% vs. <6.5%		n.s.		n.s.

^1^ Patients with T0 were excluded from this analysis. Abbreviations: OS—overall survival, DFS—disease-free survival, ECE—extracapsular extension, LNR—lymph node ratio, HR—hazard ratio, CI—confidence interval, n.s.—non significant. * Note that only SND levels I–III were included.

## Appendix C

**Table A4:** Univariate analysis of modified radical neck dissection factors associated with survival. *n* = 137.

Features	Variables	OS	DFS	Regional Control	DMFS
Gender	male vs. female	0.371	0.422	0.050	0.971
Age (years)	<65 vs. ≥65	0.011	0.128	0.853	0.275
T stage ^1^	T1–2 vs. T3–4	0.000	0.003	0.719	0.000
pN stage	pN0 vs. pN+	0.438	0.006	0.028	0.018
ECE	ECE+ vs. ECE-	0.400	0.074	0.412	0.187
HPV-status ^2^	HPV+ vs. HPV-	0.001	0.042	0.129	0.091
Previous RCT	Upfront ND vs. Salvage ND after CRT	0.301	0.708	0.388	0.694
Therapy of primary ^3^	surgery vs. CRT	0.150	0.016	0.023	0.057
Therapy of Neck (ND)	surgery vs. surgery + CRT	0.345	0.743	0.503	0.393
LNR	≥6.5% vs. <6.5%	0.010	0.002	0.073	0.003

^1^ Patients with T0 were excluded from this analysis. ^2^ Patients with unknown HPV-status were excluded from this analysis. ^3^ Surgical vs. non-surgical therapy of the primary was analyzed; cases of carcinoma of unknown primary (cT0) or isolated regional recurrences (rcT0) were excluded from this analysis. Abbreviations: OS—overall survival, DFS—disease-free survival, DMFS—distant metastasis-free survival, ECE—extracapsular extension, HPV—human papillomavirus, CRT—(chemo)radiation, LNR—lymph node ratio, HR—hazard ratio, CI—confidence interval.
